# Supplementary figures and images for: Interferon Lambda Signals in Maternal Tissues to Exert Protective and Pathogenic Effects in a Gestational Stage-Dependent Manner
Source: mBio. 2022 Apr 26;13(3):e03857-21. doi: 10.1128/mbio.03857-21 (PMC9239100; doi:10.1128/mbio.03857-21)

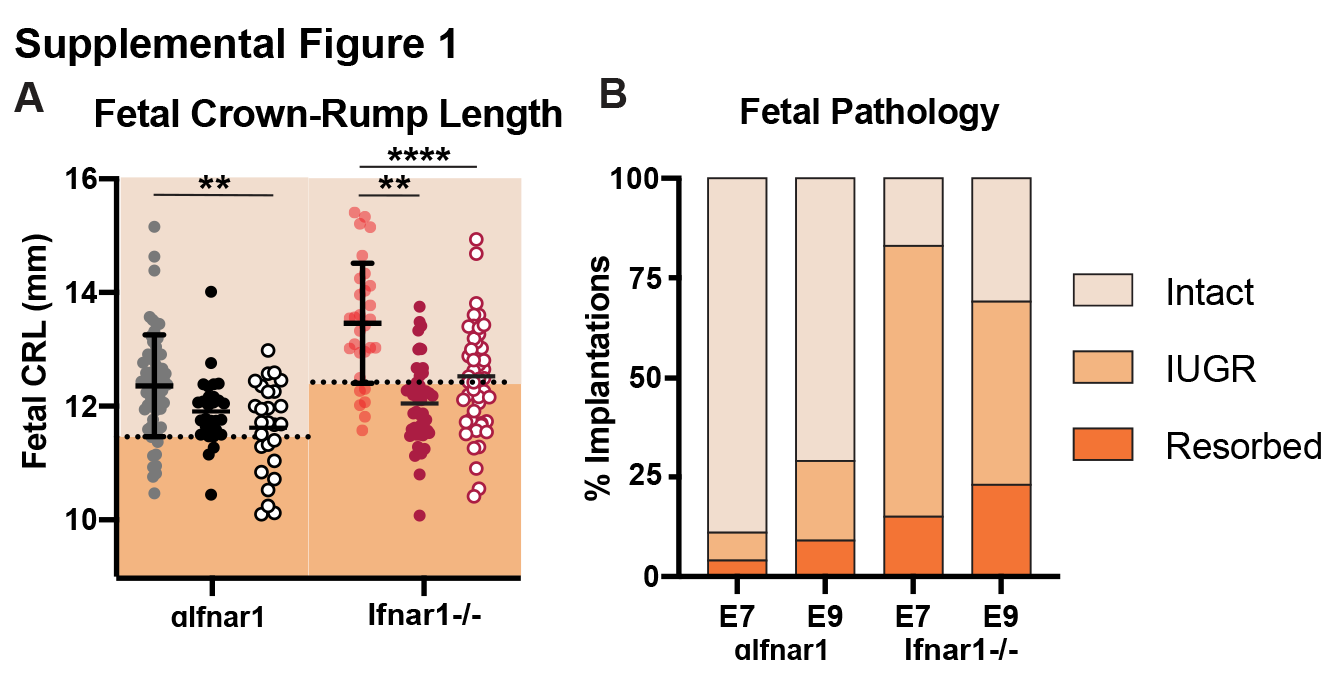

Supplement: FIG S1 [file mbio.03857-21-s0001.tif]

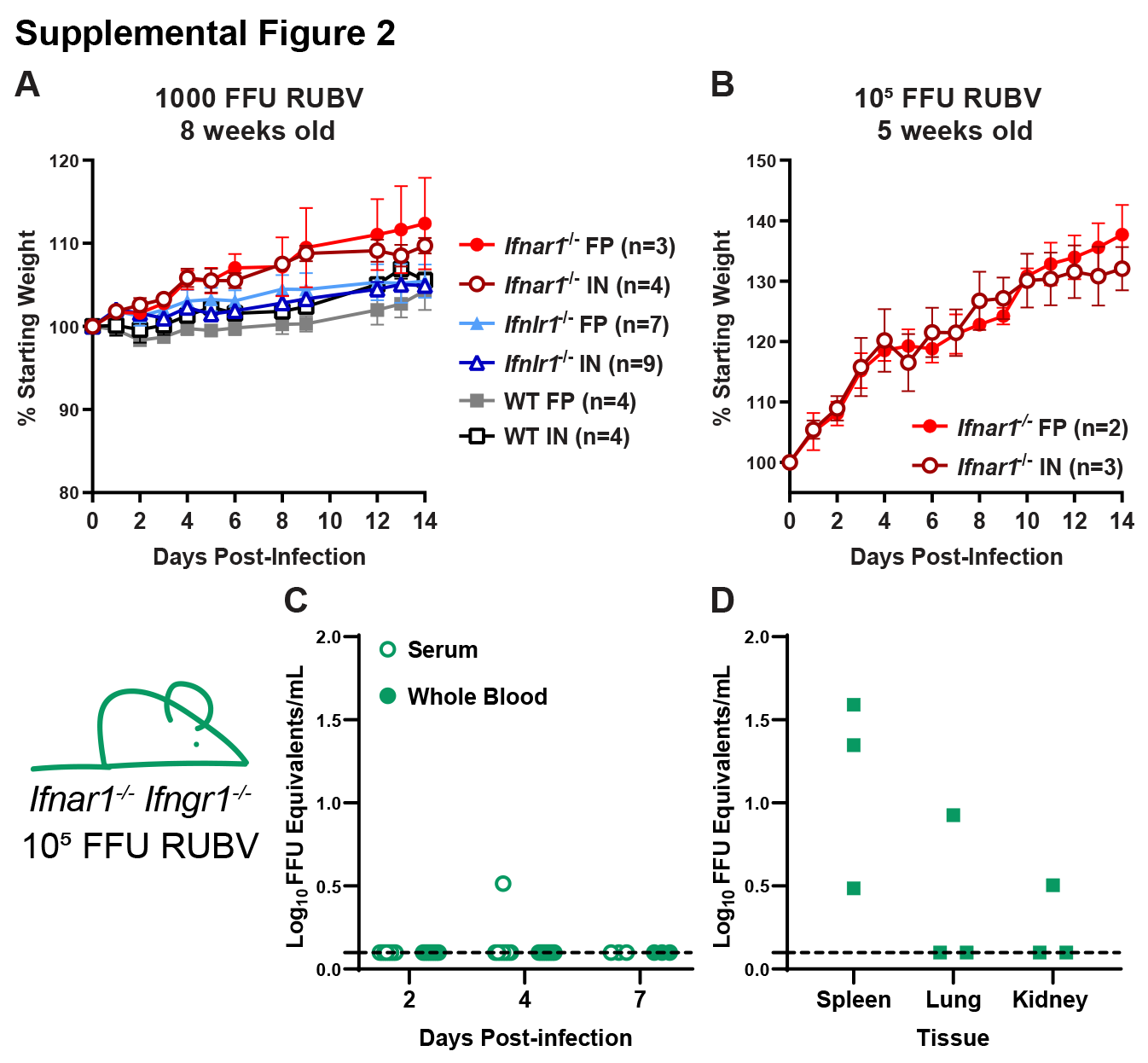

Supplement: FIG S2 [file mbio.03857-21-s0002.tif]

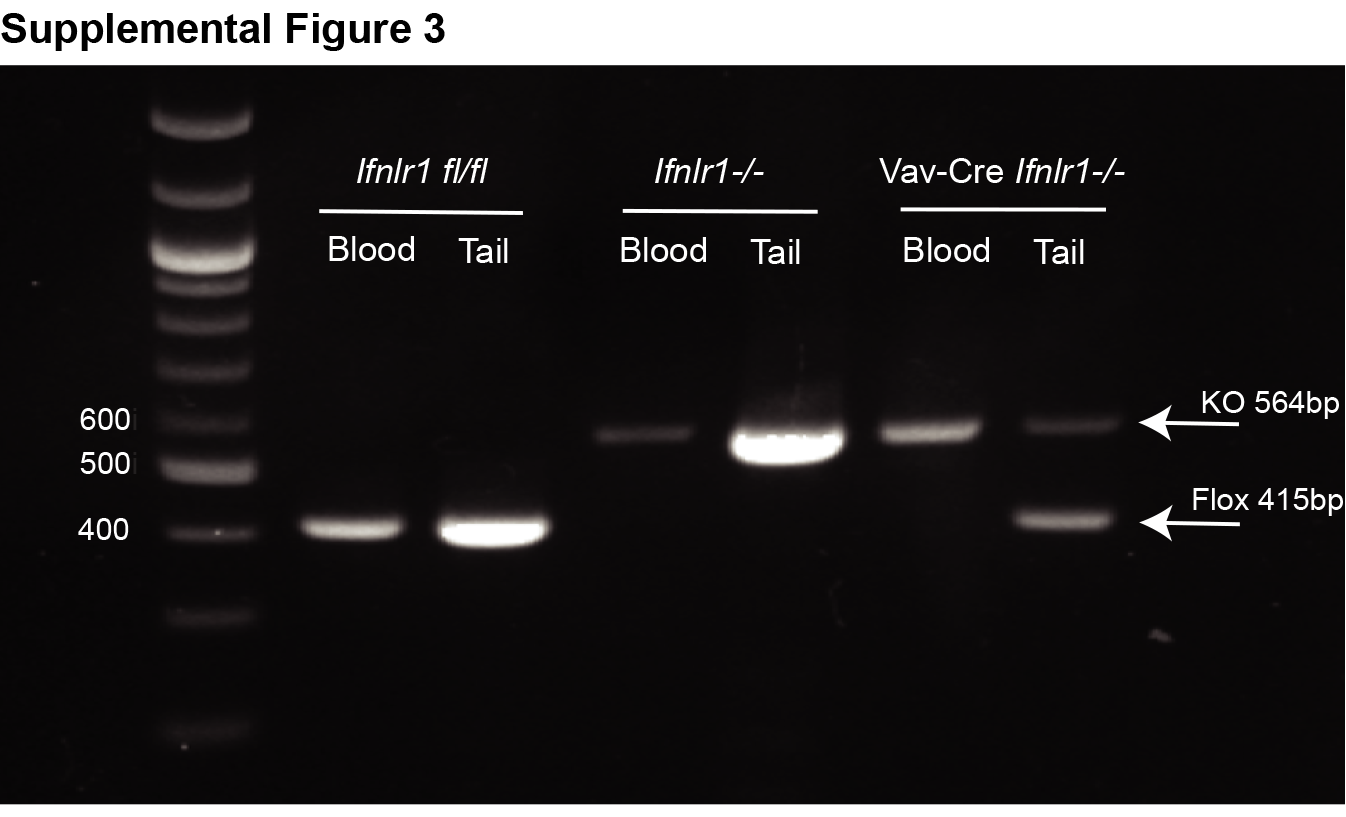

Supplement: FIG S3 [file mbio.03857-21-s0003.tif]

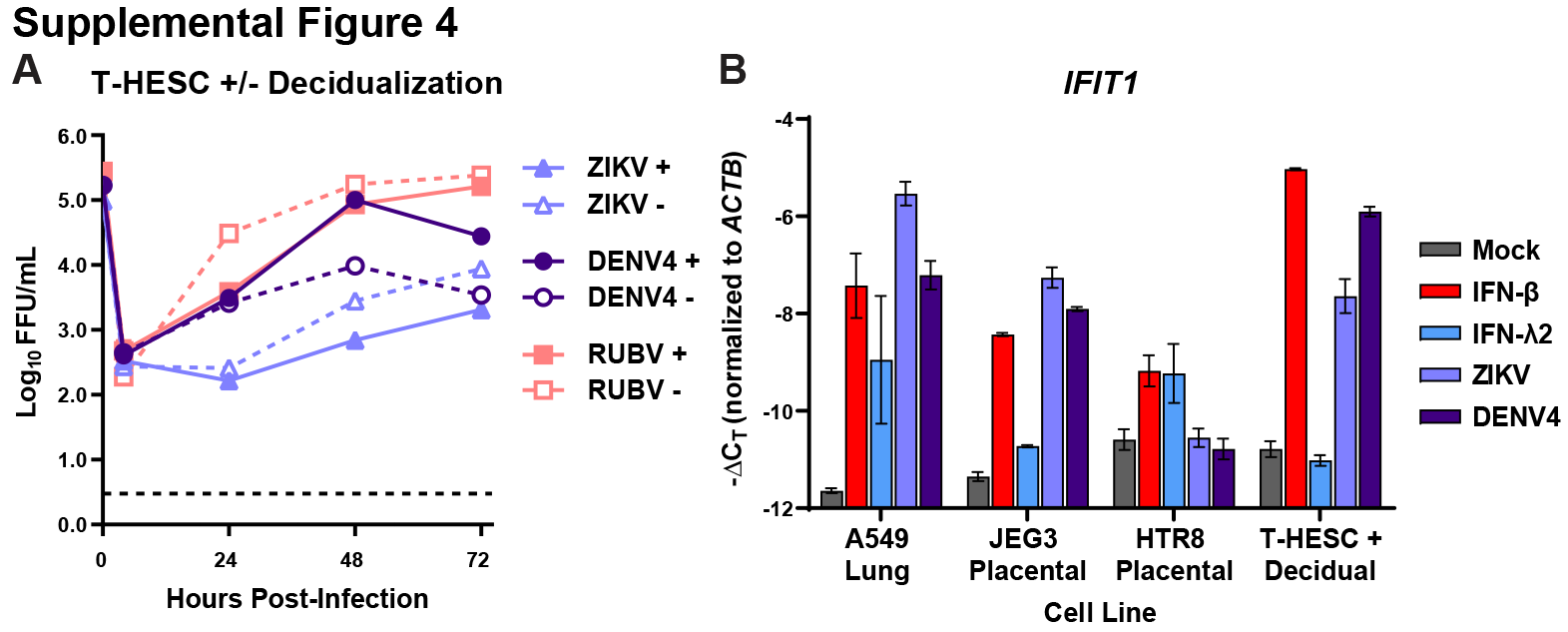

Supplement: FIG S4 [file mbio.03857-21-s0004.tif]
